# Stress erythropoiesis in atherogenic mice

**DOI:** 10.1038/s41598-020-74665-x

**Published:** 2020-10-28

**Authors:** Ángela Sánchez, Marta C. Orizaola, Diego Rodríguez-Muñoz, Ana Aranda, Antonio Castrillo, Susana Alemany

**Affiliations:** 1 Instituto de Investigaciones Biomédicas “Alberto Sols”, Consejo Superior de Investigaciones Científicas and Universidad Autónoma de Madrid, Arturo Duperier 4, 28029 Madrid, Spain; 2 Unidad de Biomedicina (Unidad Asociada Al CSIC), Universidad de Las Palmas de Gran Canaria, Las Palmas, Spain

**Keywords:** Haematopoietic stem cells, Cardiovascular diseases

## Abstract

Bone marrow erythropoiesis is mainly homeostatic and a demand of oxygen in tissues activates stress erythropoiesis in the spleen**.** Here, we show an increase in the number of circulating erythrocytes in apolipoprotein E^−/−^ mice fed a Western high-fat diet, with similar number of circulating leukocytes and CD41^+^ events (platelets). Atherogenic conditions increase spleen erythropoiesis with no variations of this cell lineage in the bone marrow. Spleens from atherogenic mice show augmented number of late-stage erythroblasts and biased differentiation of progenitor cells towards the erythroid cell lineage, with an increase of CD71^+^CD41CD34^−^CD117^+^Sca1^−^Lin^−^ cells (erythroid-primed megakaryocyte-erythroid progenitors), which is consistent with the way in which atherogenesis modifies the expression of pro-erythroid and pro-megakaryocytic genes in megakaryocyte-erythroid progenitors. These data explain the transiently improved response to an acute severe hemolytic anemia insult found in atherogenic mice in comparison to control mice, as well as the higher burst-forming unit-erythroid and colony forming unit-erythroid capacity of splenocytes from atherogenic mice. In conclusion, our work demonstrates that, along with the well stablished enhancement of monocytosis during atherogenesis, stress erythropoiesis in apolipoprotein E^−/−^ mice fed a Western high fat diet results in increased numbers of circulating red blood cells.

## Introduction

Red blood cells (RBC), like other blood lineages, develop from hematopoietic stem cells (HSC). These cells give rise to several multipotent progenitor populations (MPPs) with different potentials to develop into specific lineage-restricted progenitors. MPPs are responsible for homeostatic hematopoiesis and together with HSC are included in the so-called hematopoietic stem progenitor cell population (HSPC). They lack expression of CD38, which is upregulated in the lineage-restricted cell progenitors, which include the progenitors for all major branches of hematopoiesis: the common myeloid progenitors (CMPs) and the common lymphoid progenitors (CLPs). From the CMPs derive the granulocyte-monocyte progenitors (GMPs) and the megakaryocyte-erythroid progenitors (MEPs), which will generate the different myeloid cells and erythrocytes and platelets, respectively^1–4^.

Single-cell sequencing has revealed that these cell populations are also heterogeneous and are constituted by subsets with different potential to develop into specific cell lineages. More precisely, CD71^+^CD41^−^MEPs are strongly skewed to erythroid cell differentiation, while CD41 expression directs MEPs towards the megakaryocytic-platelets cell lineage^5–8^. CD71^+^CD41^−^MEPs progress through a series of erythroblast stages including the late-stage erythroblasts, subdivided into less mature larger basophilic immature erythroblasts (Ery. A), smaller polychromatic intermediate erythroblasts (Ery. B), and acidophilic late erythroblasts and reticulocytes (Ery. C). During the later stages, the nucleus progressively shrinks and is shed before the cells become mature RBC. Macrophages assist erythroblasts in proliferation and differentiation, and are responsible for the phagocytosis of senescent or damaged erythroid cells. CD47-CD172α cell-cell interactions. CD172α is present in the membranes of macrophages and other myeloid cells, whereas CD47 is a broadly expressed cell surface protein that acts as a marker of *self*. Recognition of this receptor inhibits erythrophagocytosis^9,10^.

Different factors can unbalance homeostatic hematopoiesis, resulting in the potentiation of specific cell lineage(s) and in the diminution of other(s). The nature of the signal will define the specific haematopoietic cell lineages that will have to adapt the generation of their corresponding differentiated cells to the new stress situation. This adaptation can occur through different mechanisms, including the amplification of specific cell populations. In adult mice, bone marrow (BM) erythropoiesis is mainly homeostatic and in situations of reduced oxygen tension there is an amplification of erythropoiesis in the spleen. Under conditions of severe hypoxia, caused by hemorrhage or BM suppression secondary to chemotherapy, there is an enormous response of the splenic stress erythropoiesis to rapidly restore the oxygen supply to tissues. This alternative stress erythropoietic pathway amplifies specific erythroid-restricted self-renewing cell progenitors in the spleen through the signalling of soluble factors, such as growth-differentiation factor 15 (GDF15), stem cell factor (SCF) and bone morphogenic protein 4 (BMP4)^11–17^. Inflammation, by infection or tissue damage, can also generate stress erythropoiesis. In response to infections, the BM cell differentiation is rapidly biased towards the myeloid branch in detriment of erythropoiesis^18,19^ along with increased levels of erythroblast apoptosis and the subsequent clearance of the apoptotic corpses (erythrophagocytosis)^20–23^. This decrease in the input of mature RBC generated in BM is compensated by splenic stress erythropoiesis (reviewed in^23,24^). In chronic inflammatory diseases the strategy to mitigate inflammatory anemia is the periodic increase of stress erythropoiesis in spleen^24–26^.

Atherogenesis is a chronic disease, arising from an increment of circulating lipids with an inflammatory component that progressively drives the expansion of circulating monocytes and the accumulation of foam cells in the arterial wall (reviewed in^27–29^). Hyperlipidemia causes oxidative stress, in which reactive oxygen species and erythrocytes have a role in the heightened harmful atherogenic processes^30–37^. Cholesterol enrichment increases HSPC proliferation^38,39^ and during the onset of atherogenesis HSPC from the BM are progressively relocated to the spleen, resulting in enhanced monocytosis not only in the BM but also in the spleen^40–42^.

In the present study we characterized the erythroid phenotype in ApoE^−/−^ mice fed a WD and showed that atherogenic mice exhibit increased number of circulating RBC. Atherogenesis promotes stress erythropoiesis in spleen with an augmented number of erythroid-primed MEPs and late-stage erythroblast cell populations, with no variations in BM erythropoiesis. These data can explain the transiently improved stress response to an acute hypoxia insult of atherogenic mice and the higher burst-forming unit-erythroid (BFU-E) and colony forming unit-erythroid (CFU-E) of splenocytes, but not of BM cells from atherogenic mice.

## Results

### Western diet increases splenic erythropoiesis in ApoE^−/−^ mice

**Figure 1:**
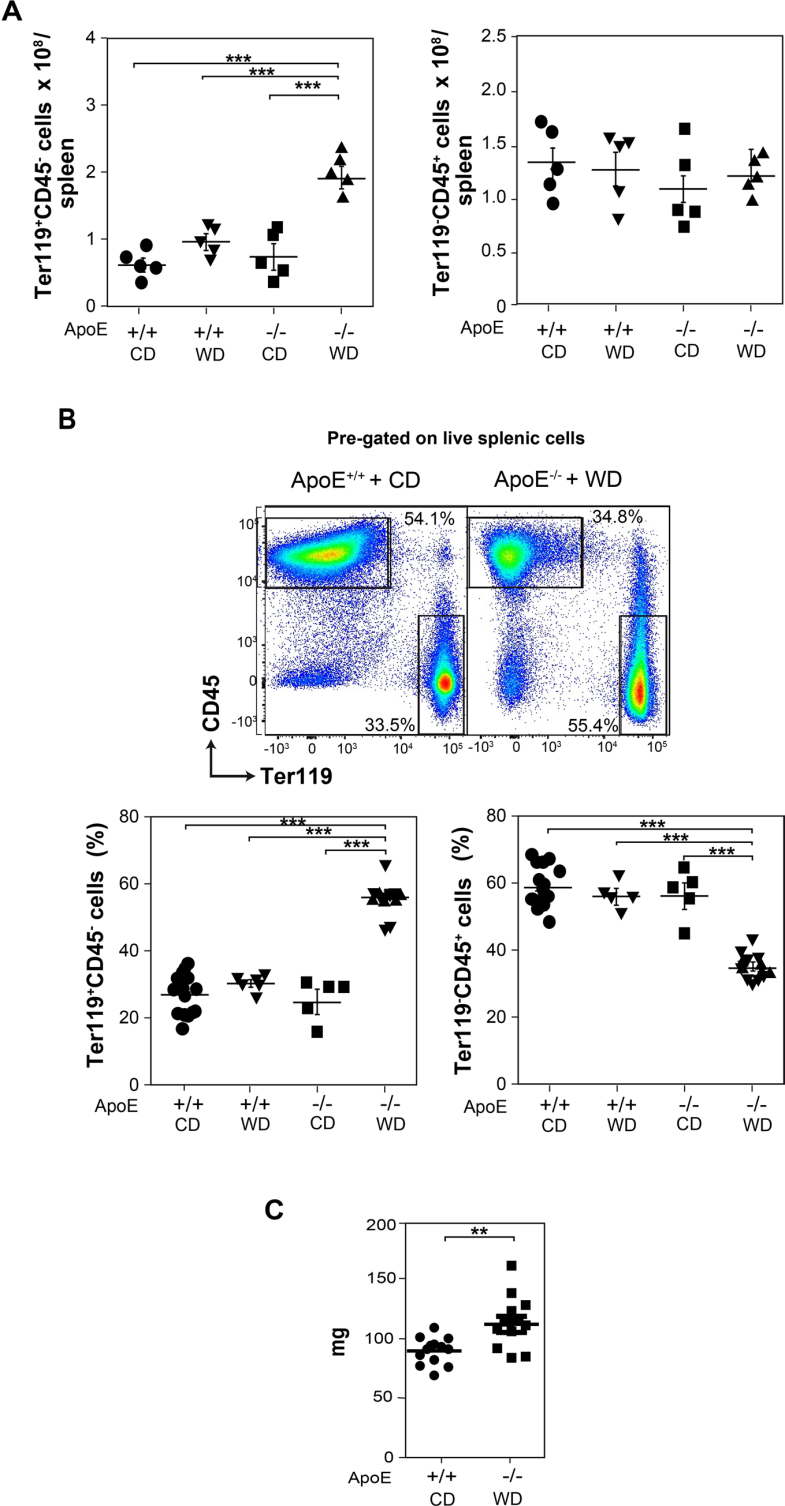
Splenic Ter119^+^CD45^−^ and Ter119^−^CD45^+^ cells in ApoE^+/+^ and ApoE^−/−^ mice fed with CD or with WD. (**A**) Number of Ter119^+^CD45^−^ and Ter119^−^CD45^+^ cells in spleen from ApoE^+/+^ and ApoE^−/−^ mice fed either with a CD or with a WD for the last 13 weeks (*n* = 5, from 3 independent experiments). (**B**) Representative graphs showing the percentage of splenic Ter119^+^CD45^−^ and Ter119^−^CD45^+^ cells in ApoE^+/+^ fed with a CD and ApoE^−/−^ mice fed with a WD, and percentage of Ter119^+^CD45^−^ and Ter119^−^CD45^+^ cells in spleens from mice described in A (n = 13–5, from 6–3 independent experiments). (**C**) Spleen weight from mice described in B. Two-tailed Student's *t*-tests were used for comparisons between the two groups (mean ± SEM; n = 13, from 6 independent experiments). **p < 0.01. (**A**,**B**) Data show the mean ± SEM. One-way ANOVA with Bonferroni correction was used to compare all pairs of columns between groups. ***p < 0.001.

Mice deficient in ApoE fed a WD rapidly develop atherogenesis^40–43^ and show increased splenic erythropoiesis (Fig. 1), determined as shown in Fig. S1A (see Supplemental Figure S1A online). In contrast with ApoE^+/+^ mice fed a chow diet (CD) or a western diet WD for 13 weeks, ApoE^−/−^ mice fed with WD for the same time period showed a significant increase in the number of late splenic erythroblasts (Ter119^+^CD45^−^ cells). This increase was not observed in ApoE^−/−^ mice fed the CD (Fig. 1A), meaning that it only occurs under hypercholesterolemic conditions (see Supplemental Table S1 online). In contrast with the erythroblasts increase, the total number of splenic leukocytes (Ter119^−^CD45^+^ cells), the other major hematopoietic cell lineage in the spleen, was not altered in ApoE^−/−^ mice fed a WD (Fig. 1A), which translated into an increased percentage of Ter119^+^CD45^−^ cells, and a reduced percentage of Ter119^−^CD45^+^ cells in this tissue (Fig. 1B). Although we and others have previously reported an increase of splenic monocytes (CD45^+^CD11b^high^F4/80^−^CD115^high^ cells) under atherogenic conditions^40–42^, these cells represent only about 1% of the total splenic cell population and their increase did not affect the total number of splenic leukocytes (see Supplemental Figure S2 online). In accordance with the expansion of Ter119^+^CD45^−^ cells, ApoE^−/−^ mice fed a WD also developed a small but significant increase in their spleen weight compared to ApoE^+/+^ mice fed a CD (Fig. 1C).

**Figure 2:**
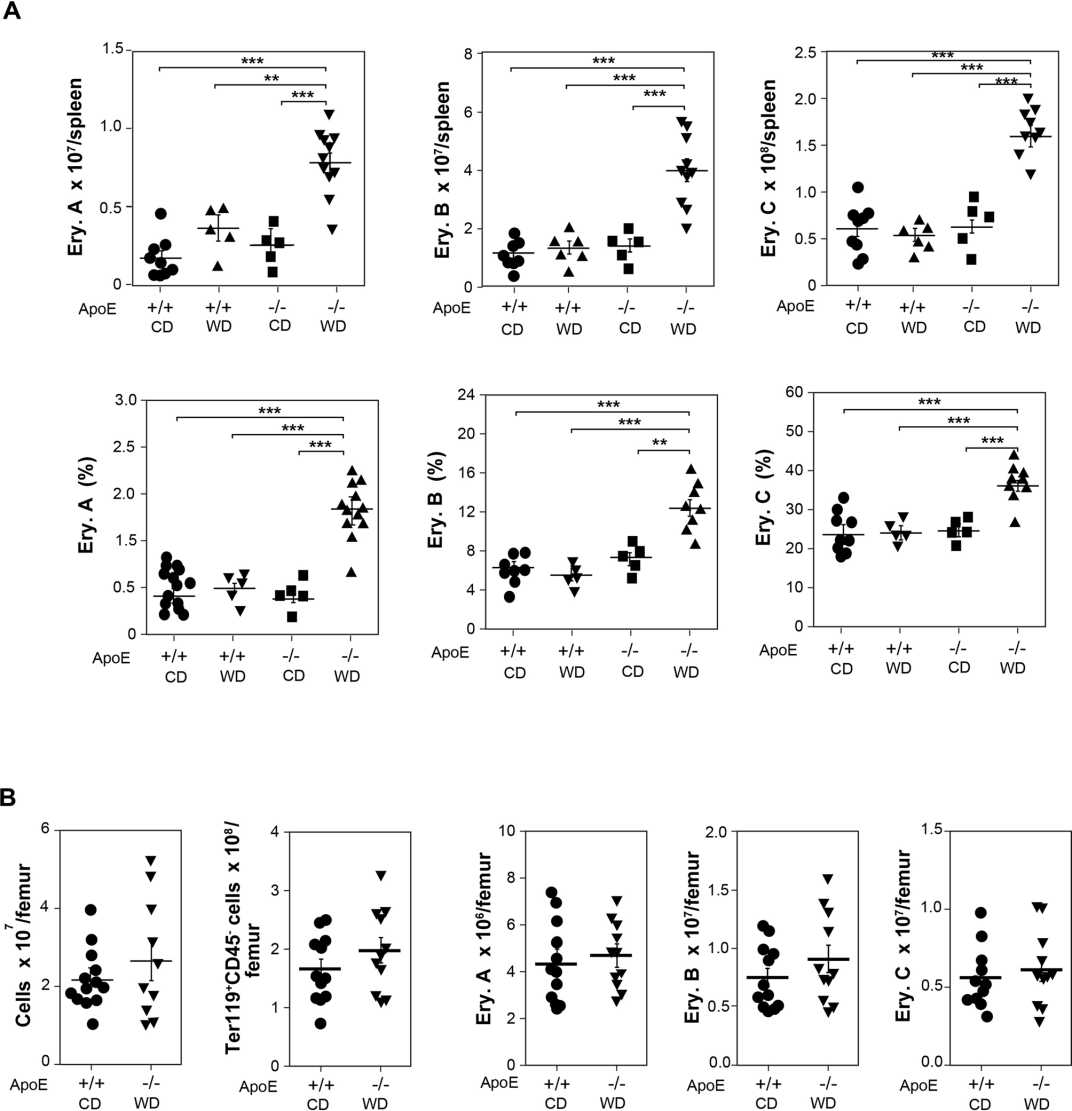
Splenic and BM Ery. A, Ery. B, and Ery. C in ApoE^+/+^ and ApoE^−/−^ mice fed with CD or with WD. (**A**) Number and percentage of Ery. A, Ery. B, and Ery. C in spleen from ApoE^+/+^ mice and ApoE^−/−^ fed either with a CD or with a WD for the last 13 weeks. One-way ANOVA with Bonferroni correction was used to compare all pairs of columns between groups (mean ± SEM; n = 13–5, from 6–3 independent experiments), **p < 0.01, ***p < 0.001. (**B**) BM cellularity, number of Ter119^+^CD45^−^ cells and of Ery. A, Ery. B, and Ery. C in BM from ApoE^+/+^ mice with a CD and ApoE^−/−^ fed with a WD for the last 13 weeks (mean ± SEM; *n* = 12–10, from 5 independent experiments).

The three late-stage erythroblasts, Ery. A, Ery. B, and Ery. C, included in the CD45^−^Ter119^+^ cell population, increased in number and percentage in the spleen of atherogenic mice (ApoE^−/−^ mice fed a WD) compared to control mice (ApoE^+/+^ mice fed a CD) and, again, ApoE deletion in conjunction with a WD was necessary to increase splenic erythroblasts (Fig. 2A). The high number of splenic late-stage erythroblasts in atherogenic mice could not be explained by a decrease in their apoptosis rate (see Supplemental Figure S3 online). Since macrophages are essential for erythroblast differentiation and senescent or damaged RBC phagocytosis^9,10^, we next analysed the possibility that changes in this cell population could underlie the increased splenic erythropoiesis in atherogenic mice. However, the number of splenic macrophages (CD11b^low^F4/80^high^) was similar in both control and atherogenic mice (see Supplemental Figure S4A online). In addition, CD172α expression on these myeloid cells, as well as CD47 expression on splenic erythroblasts, was similar in both groups of mice (see Supplemental Figure S4B,C online), which suggests similar CD47-CD172α *don’t eat me* signal interactions.

In contrast to the enhanced splenic erythropoiesis in atherogenic mice, no changes were detected in BM erythropoiesis in ApoE^−/−^ mice fed a WD respect to control mice. The number of total cells, Ter119^+^CD45^−^ cells and of the different late-stage erythroblast subpopulations in the BM were similar in both groups of mice (Fig. 2B), and even a small increase in the early apoptosis rate of these cells was observed in atherogenic mice (see Supplemental Figure S5 online).

Atherogenesis is a low-grade chronic inflammatory disease^40,42–45^ and under atherogenic conditions different pro-inflammatory cytokines transcripts, such as TNFα, IL-1β, IL-6, and IFNγ, revealed a higher expression in the spleen than in the BM, although a significant increase of IL-6 mRNA was also detected in the BM of atherogenic mice. In addition, under atherogenic conditions the mRNA levels of the anti-inflammatory cytokine IL-10 were decreased in the spleen but not in the BM (see Supplemental Figure S6A,B online). These data suggest the existence of a higher pro-inflammatory microenvironment in the spleen compared to the BM, as well as a higher expansion of the erythroid cell lineage in the spleen, but not in the BM, with an increased number of late-stage erythroblasts under atherogenic conditions.

### Atherogenic mice show increased number of circulating RBC

**Figure 3:**
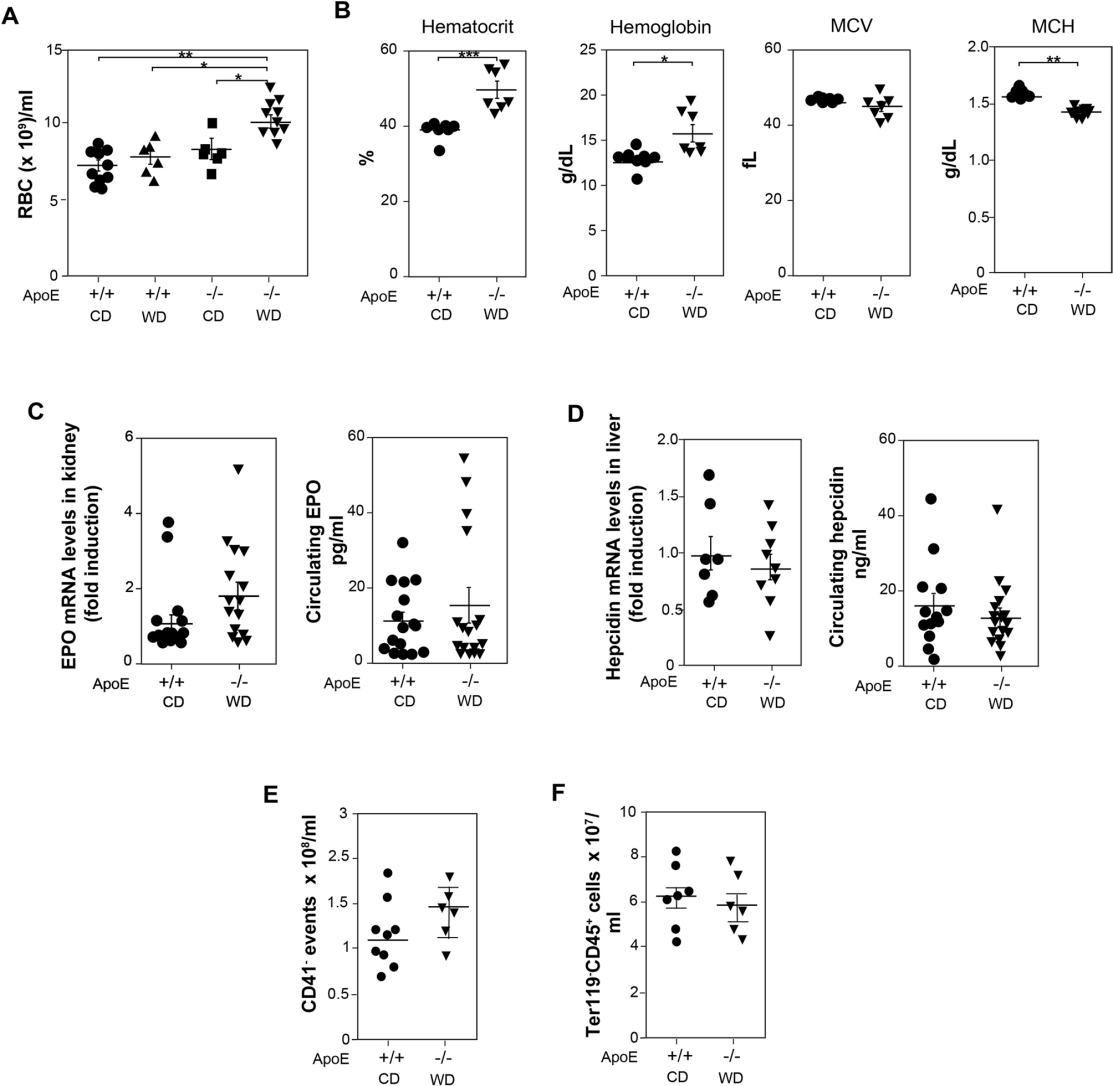
Erythroid parameters and platelets in blood from atherogenic and control mice. (**A**) Number of circulating RBC from ApoE^+/+^ and ApoE^−/−^ mice fed either with a CD or with a WD for the last 13 weeks (n = 13–6, from 6–2 independent experiments). One-way ANOVA with Bonferroni correction was used to compare all pairs of columns between groups. (**B**) Hematocrit, hemoglobin, mean corpuscular volume (MCV), and mean corpuscular hemoglobin (MCH) determined in blood from ApoE^+/+^ mice with a CD and ApoE^−/−^ fed with a WD for the last 13 weeks. Two-tailed Student's *t*-tests were used for comparisons between two groups (n = 7–7, from 3 independent experiments). (**C**) Epo mRNA levels in kidney and circulating Epo in mice described in B (n = 10–12, from 4–5 independent experiments). (**D**) Hepcidin mRNA levels in liver (n = 7–9, from 4 independent experiments) and circulating hepcidin in mice described in B (n = 7–10, from 4 independent experiments). (**E**,**F**) Number of circulating CD41^+^ events (**E**) and leukocytes (**F**) from mice described in B (n = 5–9, from 2–4 independent experiments). (**A**–**F**) Data show the mean ± SEM. (**A**,**B**) *p < 0.05, **p < 0.01.

Mature circulating RBC also express Ter119^46^ (see Supplemental Figure S7A online). ApoE^−/−^ mice fed a WD showed a higher number of circulating RBC than ApoE^+/+^ mice fed a CD, being ApoE deletion in conjunction with WD once again necessary to observe this rise (Fig. 3A). This increase in circulating RBC correlated with a higher hematocrit values under atherogenic conditions. Hemoglobin levels were slightly but significantly augmented in ApoE^−/−^ mice fed a WD compared to ApoE^+/+^ mice fed a CD. Mean corpuscular volume (MCV) value was similar in both groups of mice and mean corpuscular hemoglobin (MCH) was lower in ApoE^−/−^ mice fed WD than in ApoE^+/+^ mice fed CD, due to the more accused increase in RBC numbers, but only a mild increase of hemoglobin levels under atherogenic conditions (Fig. 3B).

Both erythropoietin (Epo) and hepcidin are major controllers of erythropoiesis. The dysregulation of these factors, among others, is behind the development of inflammatory anemia^23^. Epo hormone mediates the survival and proliferation of late-stage erythroid precursors and immature progenitors, and a decrease in the oxygen tension increases Epo production mainly in the kidney (reviewed in^47^). Similar levels of Epo mRNA in the kidney and circulating levels of Epo were detected in ApoE^−/−^ mice fed a WD and ApoE^+/+^ mice fed a CD (Fig. 3C). Hepcidin is a protein, mainly secreted be hepatocytes in the liver, whose function is to drive the degradation of the cellular iron exporter ferroportin, which restricts the iron supply to erythroid precursors^22,23^. No significant differences were detected in the hepcidin mRNA levels in the liver or in the circulation of the protein between both groups of mice (Fig. 3D). Since pro-inflammatory cytokines, mainly IL-6, increase hepcidin secretion, we determined the expression of different pro-inflammatory cytokine transcripts in liver^22^. The analysis of the pro-inflammatory microenvironment in the liver revealed a tendency towards an increased production of pro-inflammatory cytokines, being the threefold increase of TNFα transcript statistically significant (see Supplemental Figure S6C online).

In the hematopoietic hierarchy platelets and erythrocytes are closely related, deriving both from MEPs^5–8^, but ApoE^+/+^ and ApoE^−/−^ mice either fed CD or WD contained a similar number of platelets (Fig. 3E), measured as circulating CD41^+^ events (see Supplemental Figure S7B online). Similar number of circulating leukocytes was also detected in atherogenic and control mice (Fig. 3F). These data indicate that ApoE^−/−^ mice fed a WD display a higher number of circulating RBC than control mice, without a significant alteration in the circulating levels of platelets and leukocytes.

### Bias of MEPs towards the erythroid cell lineage in the spleens of atherogenic mice

**Figure 4:**
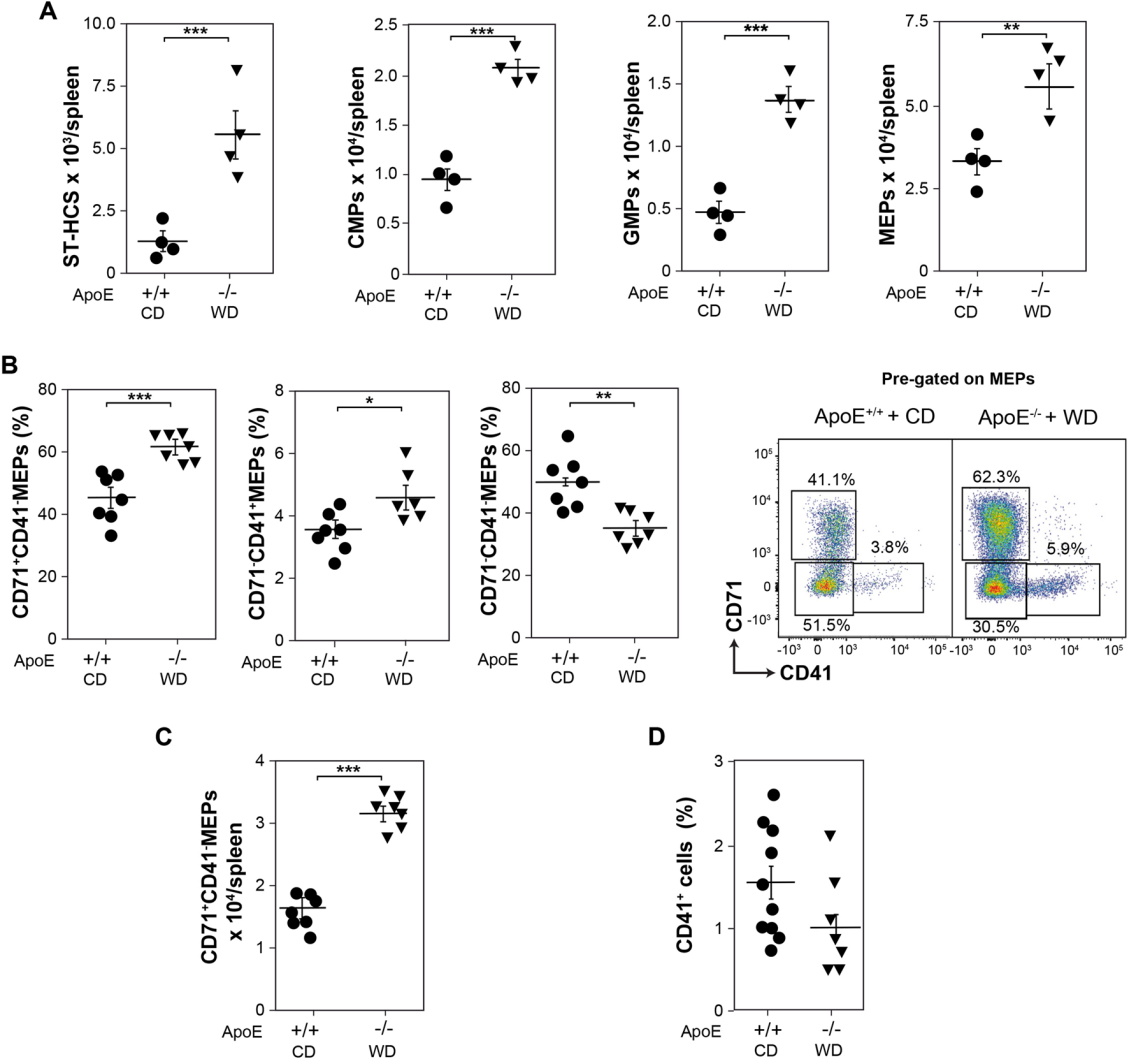
Cell progenitors in spleen from atherogenic and control mice. (**A**) Graphs showing the number of live CD34^+^LSK (CD34^+^CD117^+^Sca^+^Lin^−^) cells, CMPs (CD34^+^CD117^+^Sca1^−^CD16/32^−^Lin^−^) GMPs (CD34^+^CD117^+^Sca1^−^CD16/32^+^Lin^−^), and MEPs (CD34^−^CD117^+^Sca1^−^CD16/32^−^Lin^−^) in spleens from ApoE^+/+^ mice fed with a CD and ApoE^−/−^ mice fed with a WD for 13 weeks (*n* = 5, from 2 independent experiments). (**B**) Graphs showing the percentage of CD71^−^CD41^−^MEPs, CD71^+^CD41^−^MEPs, and CD71^−^CD41^+^MEPs respect to total splenic MEPs (n = 7, from 3 independent experiments), and a representative dot plot showing the staining of total splenic MEPs with CD71 and CD41. (**C**) Number of CD71^+^CD41^−^MEPs in spleens from mice described in A (n = 7, from 3 independent experiments). (**D**) Percentage of splenic CD41^+^ cells from mice described in A (n = 10–7, from 4 independent experiments). (**A**–**D**) Data show the mean ± SEM. Two-tailed Student's *t*-tests were used for comparisons between two groups.*p < 0.05, **p < 0.01, ***p < 0.001.

Atherogenic mice progressively relocate HSPC from the BM to the spleen, resulting in an increased number of splenic ST-HSCs CD34^+^LSK (CD34^+^Sca^+^CD117^+^Lin^−^), CMPs and GMPs, as previously determined^41,42^ (Fig. 4A). The number of MEPs, from which the erythroid and megakaryocytic cell lines derive, was also increased in the spleens of atherogenic mice compared to their control counterparts (Fig. 4A). A more detailed analysis of these splenic MEPs, gated as indicated in Fig. S8A (see Supplemental Figure S8A online), revealed a higher differentiation potential of MEPs towards the erythroid cell lineage branch in atherogenic mice than control mice. The percentage of MEPs expressing CD71 and lacking CD41 receptor (CD71^+^CD41^−^CD34^−^CD117^+^Sca1^−^Lin^−^) was augmented in the spleens of ApoE^−/−^ mice fed a WD (Fig. 4B), which correlated with the displayed two-fold increase in their number (Fig. 4C). ApoE^−/−^ mice fed a WD also showed a small increase in the percentage of splenic megakaryocytic-biased CD71^−^CD41^+^MEPs (Fig. 4B), but this was not translated into an amplification of total splenic CD41^+^ cells (Fig. 4D), determined as shown in Fig. S1B (see Supplemental Figure S1B online). These data agree with the similar number of circulating platelets in atherogenic and control mice (Fig. 3E).

The rise in the number of splenic CD71^+^CD41^−^MEPs in atherogenic mice compared to their control littermates could reflect a higher proliferative capacity of these cells. However, no differences in the distribution of cells along the different phases of the cell cycle could be detected between splenic CD71^+^CD41^−^MEPs, CD71^−^CD41^+^MEPs, and CD71^−^CD41^−^MEPs of both groups of mice (see Supplemental Figure S9A online). Furthermore, splenic Ery. A, Ery. B, and Ery. C from atherogenic and control mice showed a similar rate of Edu incorporation 4 h after its injection (see Supplemental Figure S9B online).

The increased number of splenic erythroid-biased MEPs under atherogenic conditions could also indicate a higher commitment of MEPs towards the erythroid lineage in the spleen of atherogenic mice. This commitment is controlled by a complex network of transcription factors with a decisive role in the establishment of the megakaryocytic or erythroid gene expression programmes, which mediates the differentiation of MEPs towards the megakaryocytic or erythroid cell lineages^48–56^. To better understand the mechanism by which under atherogenic conditions splenic MEPs are directed towards the erythroid cell lineage differentiation, non-biased splenic MEPs (CD71^−^CD41^−^MEPs) and erythroid-biased MEPs (CD71^+^CD41^−^MEPs) were isolated by fluorescence-activated cell sorting and the expression levels of well-known transcription factors with key roles in this process was subsequently studied. First, we determined by RT-qPCR the expression of genes that could confirm a correct separation of both types of cells. Single cell profiling has revealed higher mRNA expression of the erythroid markers Ankyrin1 and Glycophorin A in erythroid-biased CD71^+^CD41^−^MEPs opposed to non-biased CD71^−^CD41^−^MEPs^6–8^. Accordingly, splenic CD71^+^CD41^−^MEPs from both control and atherogenic mice showed higher mRNA expression of these erythroid markers than CD71^−^CD41^−^MEPs (see Supplemental Figure S10A online). Subsequently, the expressions of the transcription factor GATA1, its co-regulator FOG1, and of TAL1 (SCL), which are required for the differentiation of MEPs into both the erythroid and megakaryocyte lineages^48–52^, were analysed. Splenic CD71^−^CD41^−^MEPs and CD71^+^CD41^−^MEPs from control and atherogenic mice displayed similar mRNA levels of GATA1 and FOG1, but TAL1 mRNA levels were enhanced in splenic CD71^−^CD41^−^MEPs of atherogenic mice, reaching the expression levels observed in CD71^+^CD41^−^MEPs (see Supplemental Figure S10B online). A precise dosage of GATA2 expression is critical for early hematopoiesis, but GATA2 downregulation is necessary for the initiation of the differentiation to both the erythroid and megakaryocytic cell lineages^48,49^. Splenic CD71^+^CD41^−^MEPs of both experimental groups contained similarly decreased GATA2 mRNA levels with respect to the levels found in CD71^−^CD41^−^MEPs (see Supplemental Figure S10C online). The transcription factor RUNX1 (AML) is essential for the commitment of MEPs towards the megakaryocytic cell lineage, by the activation of the megakaryocytic gene expression programme and the repression of the erythroid master regulator EKLF (KLF1). EKLF is the crucial transcription factor that potentiates the differentiation towards the erythropoietic cell branch at the MEP bifurcation, in detriment to the megakaryocytic cell lineage^48,49,53–56^. Splenic CD71^−^CD41^−^MEPs of atherogenic mice showed significantly decreased RUNX1 mRNA compared to their control counterparts, which was even lower in CD71^+^CD41^−^MEPs of both groups of mice (see Supplemental Figure S10D online). Additionally, splenic CD71^+^CD41^−^MEPs of atherogenic mice displayed an increased level of EKLF mRNA compared to control animals. FLI1 mRNA levels, which counterbalances EKLF activity^48,49,56^ were decreased in splenic CD71^+^CD41^−^MEPs compared to CD71^−^CD41^−^MEPs, but its expression was not altered in ApoE^−/−^ mice fed a WD (see Supplemental Figure S10D online). These data indicate that atherogenic conditions modify the expression of genes involved in the specification of the transcriptional programs towards the erythroid or megakaryocytic cell lineages in splenic MEPs, favouring their differentiation towards the erythroid lineage.

### Erythropoietic stress response in ApoE^−/−^ mice fed a WD

**Figure 5:**
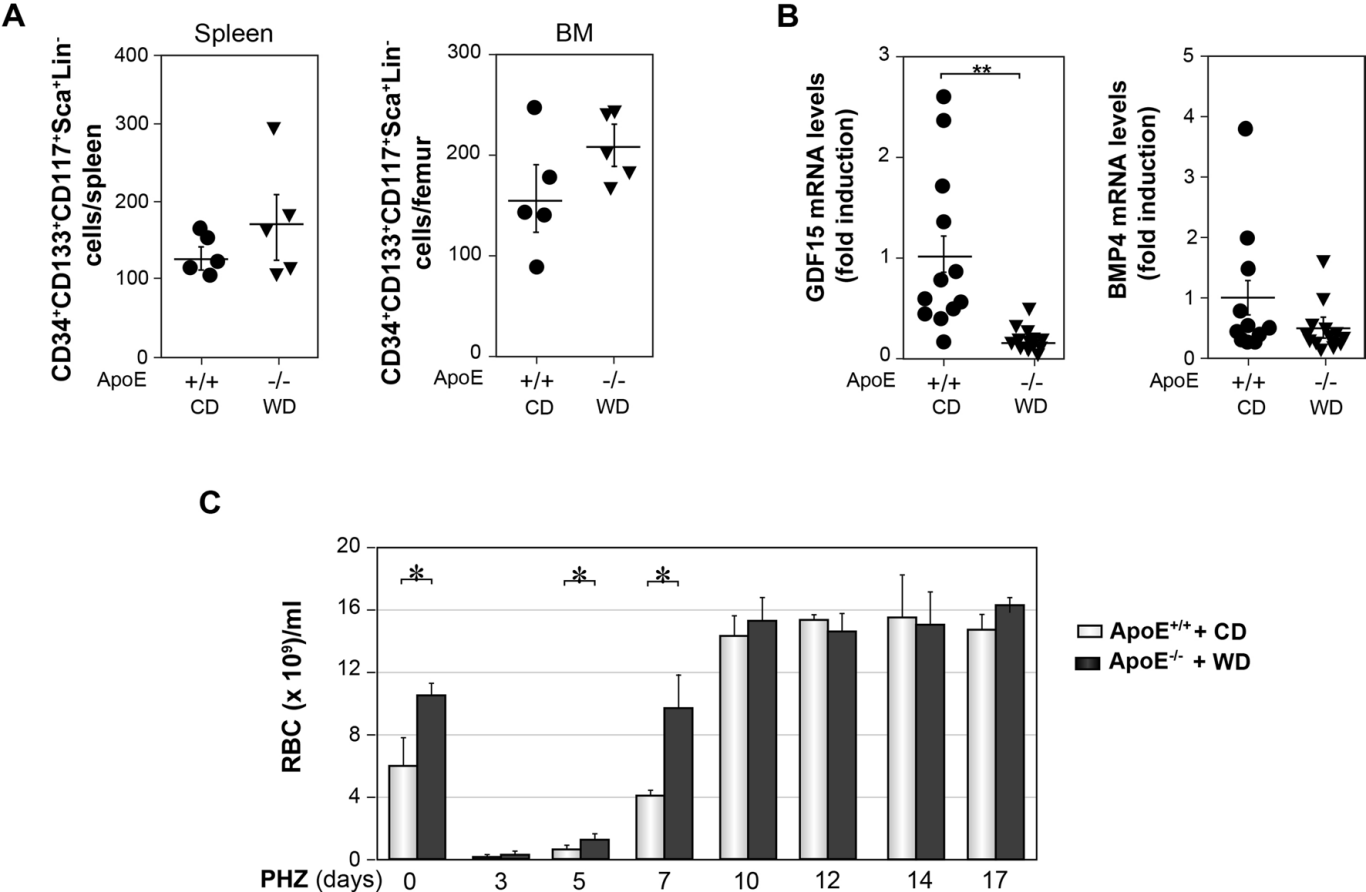
Stress erythropoiesis in control and atherogenic mice. (**A**) Graphs showing the number of live CD34^+^CD133^+^CD117^+^Sca1^+^Lin^−^ cells in the spleen and BM from ApoE^+/+^ mice fed with a CD and ApoE^−/−^ mice fed with a WD for 13 weeks (*n* = 5, from 3 independent experiments). (**B**) GDF15 and BMP4 mRNA levels in the spleen from mice described in A (n = 12, from 4–5 independent experiments). (**C**) Circulating RBC in mice described in A injected with 100 mg/kg mouse of PHZ (*n* = 7–8, from 2 independent experiments). Data show the significant differences between atherogenic and control mice within the same day. (**A**–**C**) Data show the mean ± SEM. (**B**,**C**) Two-tailed Student's *t*-tests were used for comparisons between two groups *p < 0.05, **p < 0.01.

As occurs under atherogenic conditions^41,42^, a hallmark of stress erythropoiesis is the migration of HSPC from the BM to the spleen^11,12^. In response to a low oxygen tensión a specific erythroid-restricted self-renewing progenitor (CD34^+^CD133^+^CD117^+^Sca1^+^Lin^−^) cell population is greatly amplified in the spleen, which supports the rapid production of new circulating RBC. GDF15, partly mediated by its capacity to increase splenic BMP4 levels, promotes their maintenance, proliferation, and differentitation in this organ^13–15,17^. Similar numbers of these specific erythroid progenitor cells, determined as indicated in Figure S8B (See Supplemental Figure S8B online), were detected in the spleen and the BM of ApoE^−/−^ mice fed a WD with respect to the ApoE^+/+^ mice fed a CD (Fig. 5A). Surprisingly, the spleens of atherogenic mice after 13 weeks with WD, displayed a decrease in the expression levels of GDF15 transcript compared to control mice, but no significant differences between both groups of mice were detected respect to the mRNA expression of BMP4 (Fig. 5B).

Phenylhydrazine (PHZ) is a toxic agent, that induces acute severe hemolytic anemia in mice, which is rapidly compensated by the deployment of an enormous splenic stress erythropoietic response ^11,12,14,15^. We next evaluated the responsiveness of control and atherogenic mice to this severe acute hypoxia insult. Both types of mice were intraperitoneally injected with a single dose of 100 mg/kg mouse of PHZ^14^, which induced a similar decrease (more than 95%) in the number of circulating RBC on day 3 post PHZ-injection. On days 5 and 7 post-treatment, atherogenic mice showed a higher number of circulating RBC than control mice. However, the concentration of circulating RBC reached a plateau on day 10 post PHZ-injection, with similar values for both groups of mice (Fig. 5C). In conclusion atherogenic and control mice show similar levels of CD34^+^CD133^+^CD117^+^Sca1^+^Lin^−^ cells, at the time tested. However, ApoE^−/−^ mice fed a WD show a further transiently improved stress erythropoietic response to the PHZ-induced acute severe hypoxia insult, compared to control mice.

### Increased erythroid-colony forming capacity of splenocytes from atherogenic mice

**Figure 6:**
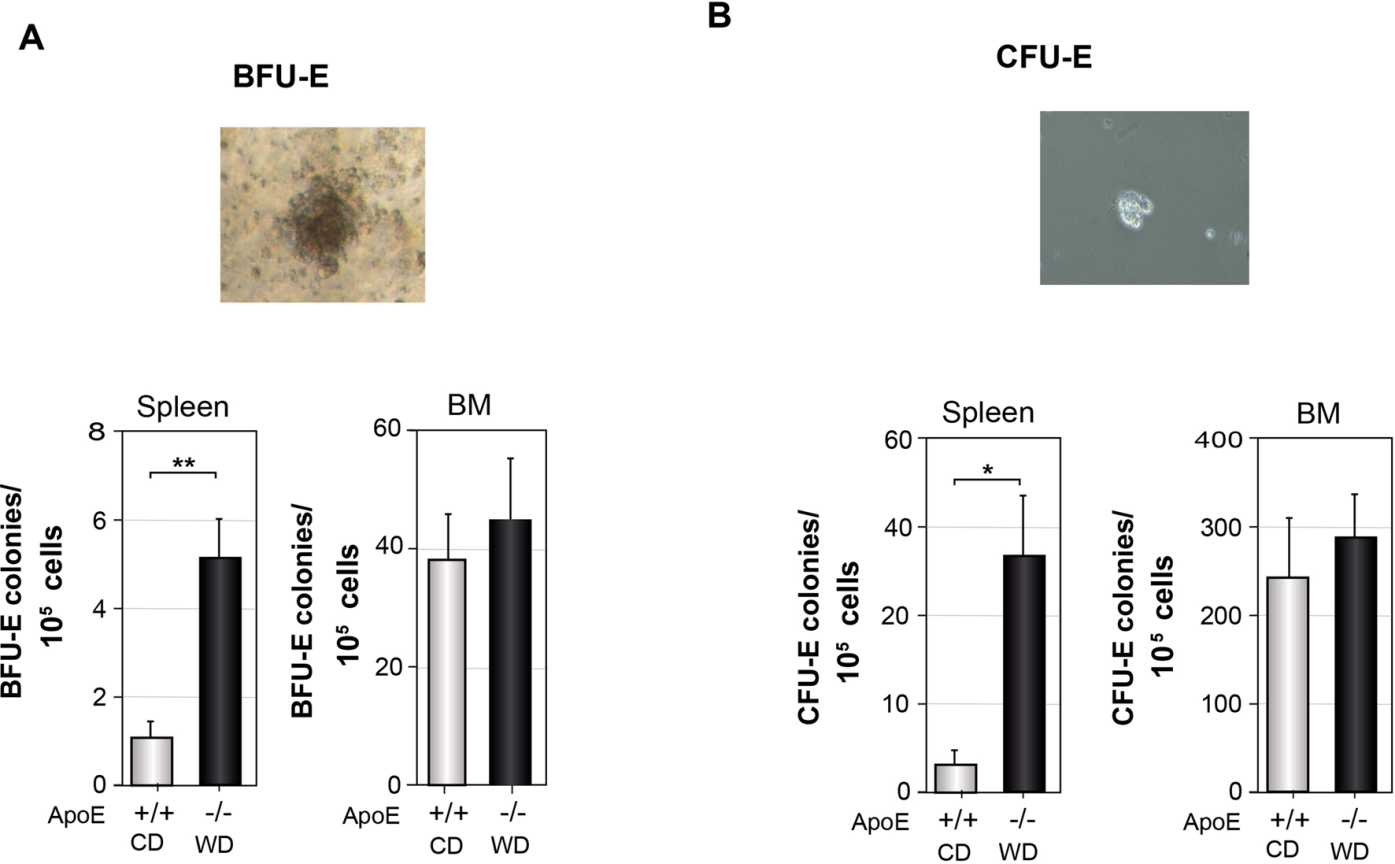
Erythroid-colony forming assays of splenocytes and BM cells from control and atherogenic mice. Splenocytes and BM cells from ApoE^+/+^ fed with a CD and ApoE^−/−^ mice fed a WD for the last 13 weeks were subjected to BFU-E and CFU-E analysis as described under “Materials and methods”. (**A**) BFU-E morphological characteristics (upper panel) and BFU-E counted after 9 days of incubation (lower panels). (**B**) CFU-E morphological characteristics (upper panel), CFU-E progenitors from mice described in (**A**) counted after 2 days of incubation (lower panels). (**A**,**B**) Data show the mean ± SEM, n = 4, triplicate in each assay. Two-tailed Student's *t*-tests were used for comparisons between two groups.*p < 0.05, **p < 0.01.

The earliest committed erythroid progenitors identified ex vivo are the BFU-E and the further differentiated CFU-E, two populations of cells with modifiable self-renewal, proliferation and differentiation capabilities (reviewed in^57^). Stress erythropoiesis increases BFU-E and CFU-E in the spleen^12,17,24^. We carried out erythroid-colony forming assays with hypotonic lysed splenocytes and BM cells from ApoE^−/−^ mice fed a WD and ApoE^+/+^ mice fed a CD. The number of splenic BFU-E from atherogenic mice increased 500% respect to control mice levels, whereas BM cells from both groups showed similar BFU-E (Fig. 6A). Furthermore, splenocytes from ApoE^−/−^ mice fed a WD generated about 10 times more CFU-E than the ones from control mice. BM cells from atherogenic and control mice showed similar CFU-E (Fig. 6B). These data indicate that splenocytes, but not BM cells, from atherogenic mice display a higher erythroid-colony forming capacity, a hallmark of stress erythropoiesis.

## Discussion

It is well documented that under atherogenic conditions the generation of monocytes increases in both the BM and the spleen^40–43^. We show here that ApoE^−/−^ mice fed a WD also display increased stress erythropoiesis in the spleen, which results in a higher number of circulating RBC compared to their control counterparts (ApoE^+/+^ mice fed a CD). The analysis of circulating hematopoietic cells in Ldr^−/−^ mice fed with WD or with CD, has also determined an increase in the absolute number of circulating RBC under atherogenic conditions^43^. These data indicate that in mice the deletion of either ApoE or Ldr together with high-fat nutrition, results in an increment of the number of circulating erythrocytes. In addition to this, in atherogenic Ldr^−/−^ mice, GMPs show an increased expression of pro-monocytic lineage genes, inducing their bias towards the monocytic lineage-commitment, contributing to the development of monocytosis under hypercholesterolemic conditions^43^. Our results show an amplification of the erythroid population pool in the spleens of ApoE^−/−^ mice fed a WD, with no significant variations in the apoptosis or proliferation rate of splenic MEPs and late-stage erythroblasts. However, there is an increase in the absolute number of splenic, late-stage erythroblasts and erythroid-primed MEPs, which is consistent with the way in which the expression of genes (TAL1, RUNX1, and EKLF1) involved in megakaryocytic-erythroid bifurcation is altered in splenic MEPs of ApoE^−/−^ mice fed a WD.

Infections and tissue damage processes that course with inflammation promote a biased BM hematopoiesis towards the myeloid cell lineage, in order to confront the immune distress^18,19^. Furthermore, erythroid precursors generated in the BM under these circumstances display a higher apoptosis rate, and their fully differentiated RBC present a lower half-life than normal^20,21,23^. These events translate into a reduced number of circulating RBC. To alleviate this inflammatory anemia, the spleen assumes the main erythropoietic role, and stress erythropoiesis is induced in order to meet the oxygen demand in the organism. In chronic inflammatory processes periodic waves of stress erythropoiesis occur^24–26^.

Atherogenesis is a low-grade chronic inflammatory disease^40,42–45^ with an enlarged production of cytokines in different tissues, as also shown here. In this context, the increase of the harmful oxygen-dependent processes, related to erythropoiesis, further enhance inflammation^30–37^. Atherogenesis promotes splenic erythropoiesis with no significant variations in BM erythropoiesis, resulting in a higher concentration of circulating RBC. In contrast to other inflammatory pathologies, atherogenesis does not course with leucocytosis as shown here and as previously determined^43^. In addition, the platelet cell lineage, which is closely related to the erythropoietic lineage in the hematopoietic hierarchy, shows no significant variation between atherogenic and control mice, as determined by the number of circulating CD41^+^ events.

Splenic stress erythropoiesis is dependent on the migration capacity of HSPC to the spleen where the amplification of the erythropoietic cell lineage takes place. The mechanisms involved in this process are not totally coincident with those implicated in steady state erythropoiesis (reviewed in^12,24^). In agreement with previous reports^41,42^, we show here an increased number of splenic ST-HSC in atherogenic mice. Although we have not detected, at the time tested, a higher number of CD34^+^CD133^+^CD117^+^Sca1^+^Lin^−^ cells in the spleen, our data show an increase in the number of splenic erythroid-primed MEPs (CD71^+^CD41^−^CD34^−^CD117^+^Sca1^−^Lin^−^). These data agree with the improved response to the acute hemolytic anemia induced by PHZ in atherogenic mice, as well as with the higher BFU-E and CFU-E of the splenocyte population in these mice.

In conclusion, in addition to the well-established monocytosis enhancement, our results demonstrate that atherogenic conditions promote the activation of splenic stress erythropoiesis in mice, resulting in an increased number of circulating RBC.

## Materials and methods

### Animals, diets, phenylhydrazine treatment

ApoE^+/+^ and ApoE^−/−^ C57BL/6J mice were maintained in the animal house in accordance with the Institutional Guidelines for the Care and Use of Laboratory Animals in Research and the Relevant European Council Directive (2010/63/EU) and Spanish law (R.D. 1201/2005). All the methods were carried out in accordance with the relevant guidelines and regulations, and with approval of the Ethics Committee of the Consejo Superior de Investigaciones Científicas and of the Ethics Committee of the Comunidad de Madrid (PROEX090/15). Male mice were fed a normal CD throughout or were switched to a WD (S9167-E012, high fat + 7.5 g/kg cholesterol, Sniff Spezialdiäten GmbH,) at 10 weeks of age and maintained on this WD for 13 weeks, as previously described^40^. The animals were then killed by CO_2_ inhalation. To generate acute severe hypoxia^14^, mice were intraperitoneally injected with 100 mg/kg mouse of phenylhydrazine (114715, Sigma-Aldrich). Starting the day of the treatment, blood was collected on alternating days and subsequently subjected to FACS analysis.

### Cell isolation and flow cytometry analysis

Blood, BM cells and splenocytes were collected as previously described^19,40^. The different cell samples (0.5–2 × 10^6^ cells) were stained for the indicated surface markers (see Supplemental Table S2 online) for 20 min at room temperature and subsequently washed twice with buffer A. We distinguished live and dead cells by adding SYTOX Green (S7020, Life Technologies) or DAPI (D9542, Sigma-Aldrich) 5 min before FACS analysis. Haematopoietic progenitors were isolated from the spleen and BM by magnetic-activated cell sorting (MACS) using a Lineage Cell Depletion Kit (130-090-858, Miltenyi) and MACS separation MS columns (130-042-201, Miltenyi) prior staining. Unstained cells were used as a negative control to establish the flow cytometer voltage settings, and single-colour positive controls were used to adjust compensation. The absolute number of cells was calculated by adding Perfect-Count Microspheres (CYT-PCM-100, Cytognos) to the flow cytometry samples. Apoptosis, cell cycle, and cell proliferation were determined by flow cytometry as previously described^19,40^. The flow cytometry data were acquired using a FACSCanto II and analysed with FACSDiva (Becton and Dickinson) or FlowJo software.

### Cell sorting and quantitative real-time PCR assays

Splenic lineage negative (Lin^−^) cells were isolated, stained and sorted as previously described^19^. Approximately 500 events were collected and RT-qPCR analysis of specific genes (see Supplemental Table S3 online) was performed using the CellsDirect™ One-Step RT-qPCR Kit (11753100, ThermoFisher). Total RNA isolated from spleen, kidney, and liver was used to perform RT-qPCR assays as described previously^58^.

### Blood analysis

To obtain plasma, blood was collected using EDTA as anticoagulant and samples were centrifuged for 10 min at 2000×*g* within 30 min of collection. The Hepcidin Mouse ELISA Kit (E4693-100, BioVision) was used for in vitro quantitative determination of hepcidin and the Legend Max™ Mouse EPO ELISA Kit (442707, Biolegend) was used for the quantitative determination of Epo. Cholesterol circulating levels were measured by using commercial kits for total cholesterol (10745065, Reflotron, Roche Diagnostics, Mannheim Germany). Hemogram was performed at DYNAMIMED SL. Parque Científico (Madrid).

### Colony assays

BM cells and splenocytes were collected and treated with hypotonic buffer as previously described^19,40^, plated at different densities in p12-wells and incubated at 37 °C in an atmosphere containing 5% CO_2_. For the quantification of BFU-E, methylcellulose-based semi-solid culture medium MethoCult™ SF M3436 (Stem Cell Technologies) was used, following the manufacturer’s instructions. After 9 days of incubation colonies were counted in an inverted microscope at a 10× magnification. For detection of CFU-E, the methylcellulose-based medium MethoCult™ M3334 (Stem Cell Technologies) was used, following the manufacturer’s instructions. After 48 h of incubation, CFU-E were counted in an inverted microscope at 20× magnification.

### Statistical analysis

The results are expressed as means ± SEM. Two-tailed Student's *t*-tests were used for comparisons between two groups. For comparisons among more than two groups, one-way ANOVA with Bonferroni correction was used to compare all pairs of columns between groups. Differences between the groups were considered significant if: *p < 0.05, **p < 0.01, or ***p < 0.001.

## Supplementary information

Supplementary Information.
