# Supplementary material for: Stress erythropoiesis in atherogenic mice
Source: Sci Rep. 2020 Oct 28;10:18469. doi: 10.1038/s41598-020-74665-x (PMC7595174; doi:10.1038/s41598-020-74665-x)
Supplement: Supplementary file 1 — Supplementary Information. [file 41598_2020_74665_MOESM1_ESM.pdf]

Supplemental Data

Stress erythropoiesis in atherogenic mice

Ángela Sánchez, Marta C. Orizaola, Diego Rodríguez, Ana Aranda, Antonio Castrillo, and Susana Alemany*

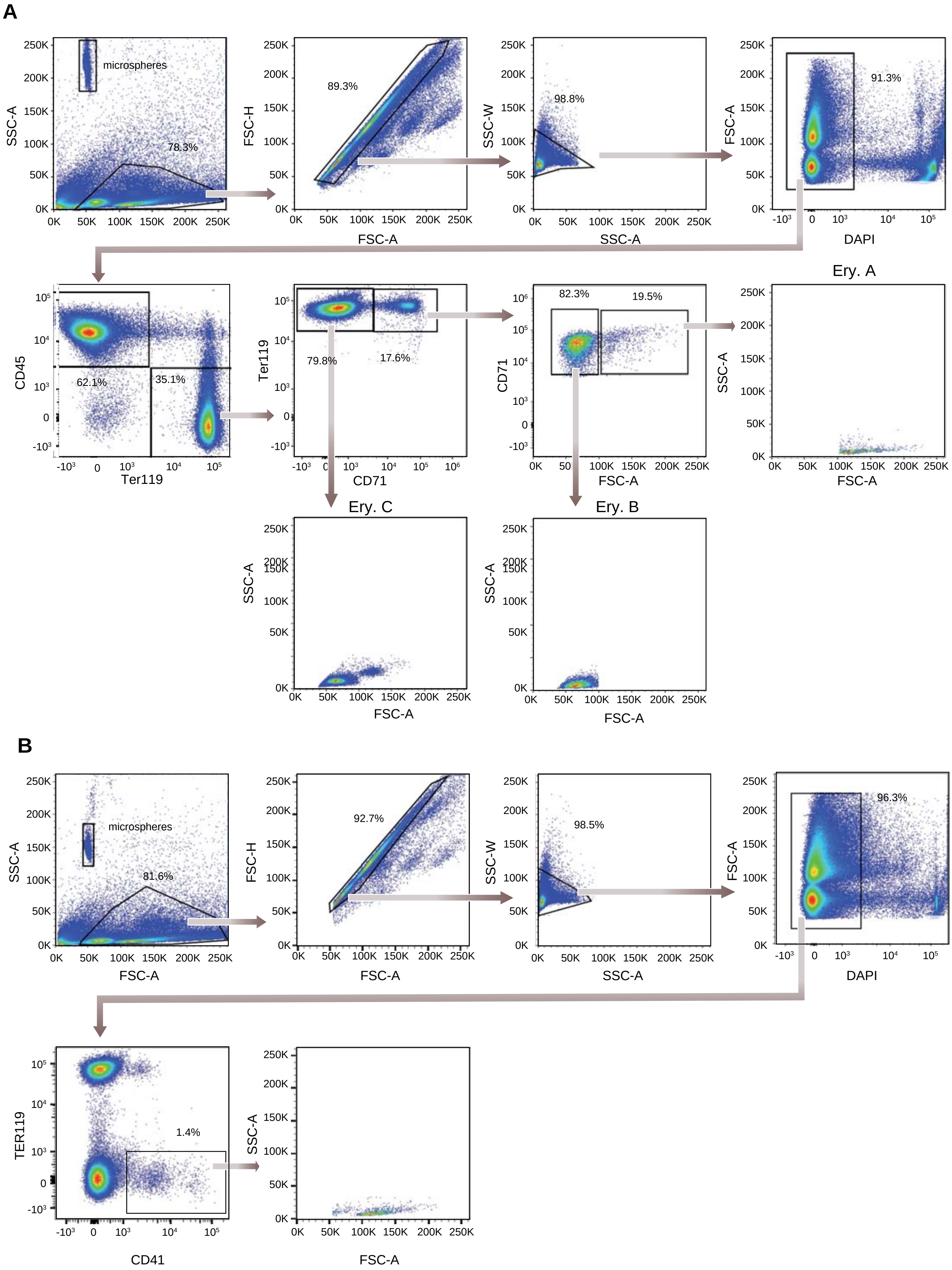

Supplementary Figure 1. Gating strategy for Ter119^{+}CD45^{-} and Ter119^{-}CD45^{+} cells, Ery. A, Ery. B, Ery. C, and CD41^{+} cells. A) Single live splenic cells were plotted for CD45 and Ter119. CD45^{-}Ter119^{+} cells were then split into subgroups based on their expression of CD71. Ter119^{+}CD71^{-} cells were identified as Ery. C, and Ter119^{+}CD71^{+} cells were further subdivided based on their size. Ter119^{+}CD71^{+}FCS^{low} cells were identified as Ery. B, and Ter119^{+}CD71^{+}FCS^{high} cells as Ery. A. **B)** Single live splenic cells were plotted for CD41 and Ter119.

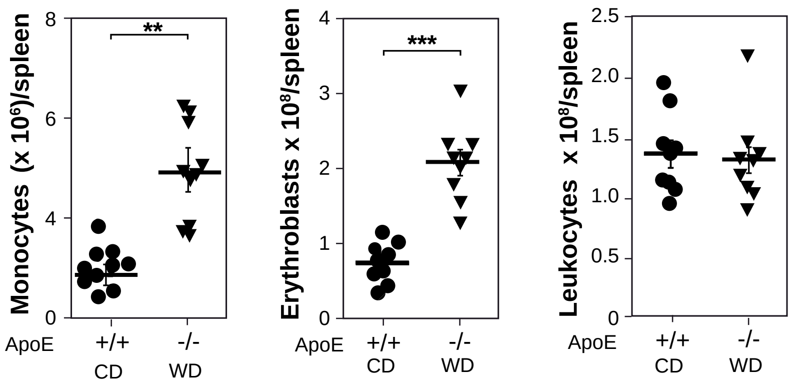

Supplementary Figure 2. Splenic monocytes, late erythroblasts and leukocytes in control and atherogenic mice. Number of monocytes ($CD45^{+}CD11b^{high}F4/80^{-}CD115^{high}$), late erythroblasts **(**$Ter119^{+}CD45^{-}$) and leukocytes ($Ter119^{-}CD45^{+}$) cells in the spleen of $ApoE^{+/+}$ mice fed a CD and $ApoE^{-/-}$ mice fed a WD for 13 weeks (*n*=9, from 4 independent experiments). Data show the mean ± SEM. Two-tailed Student's *t*-tests were used for comparisons between two groups. ***p*<0.01, ****p*<0.001.

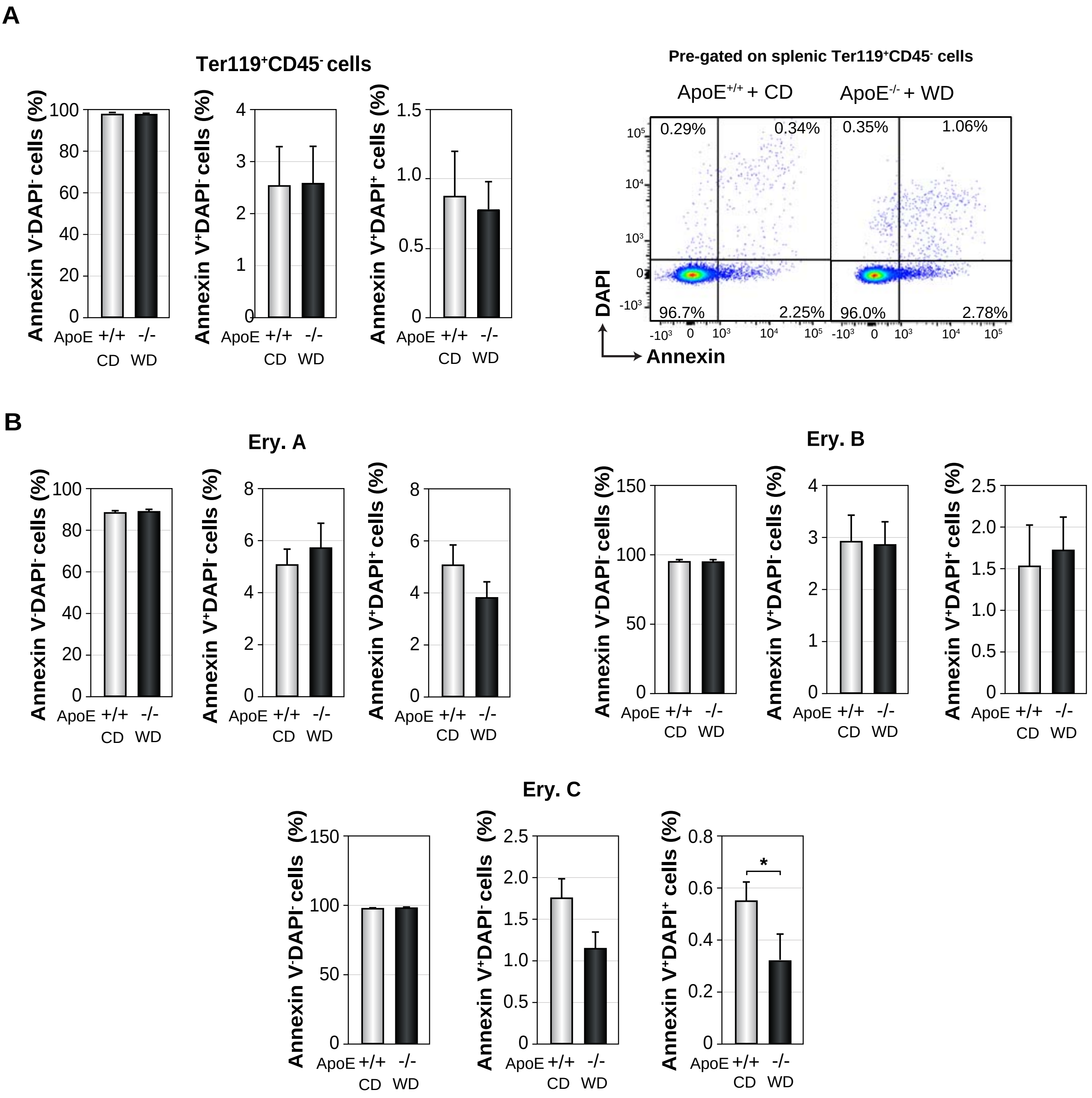

Supplementary Figure 3. Analysis of apoptosis among splenic Ter119$^+$CD45$^-$ cells, Ery. A, Ery. B, and Ery. C from control and atherogenic mice. A) Percentage of Annexin V$^-$DAPI$^-$, Annexin V$^+$DAPI$^-$ and Annexin V$^+$DAPI$^+$ cells among splenic CD45$^-$Ter119$^+$ cells from ApoE$^{+/+}$ mice fed a CD and ApoE$^{-/-}$ mice fed a WD for 13 weeks. The right panel depicts a representative dot plot showing the staining of cells with Annexin V and DAPI. **B)** Percentage of Annexin V$^-$DAPI$^-$, Annexin V$^+$DAPI$^-$ and Annexin V$^+$DAPI$^+$ cells among splenic Ery. A, Ery. B, and Ery. C. Graphs show means ± SEM (*n*=6, from 3 independent experiments). Two-tailed Student's *t*-tests were used for comparisons of two groups. *p<0.05.

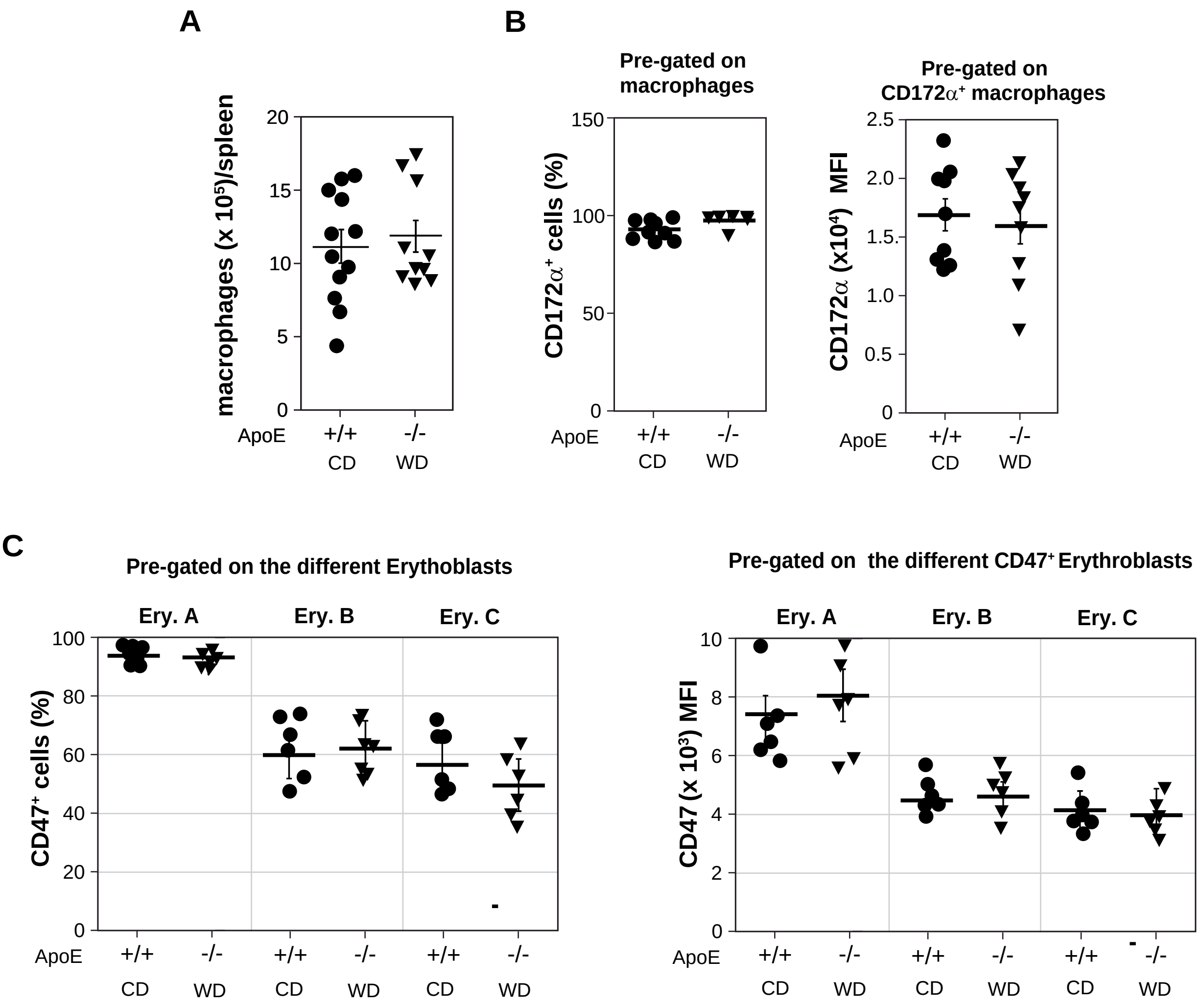

Supplementary Figure 4. Expression of CD172α on splenic macrophages and expression of CD47 on splenic Ery. A, Ery. B, and Ery. C from control and atherogenic mice. A) Number of splenic macrophages ($CD11b^{low}F4/80^{high}$) from ApoE$^{+/+}$ mice fed with a CD and ApoE$^{-/-}$ mice fed with a WD for 13 weeks (n=9, from 4 independent experiments). **B)** Percentage of splenic macrophages from mice described in A positive for CD172α and mean fluorescence intensity (MFI) of CD172α on these cells (*n*=9-7, from 4-3 independent experiments). **C)** Percentage of splenic Ery. A, Ery. B, and Ery. C positive for CD47 from mice described in A, and MFI of CD47 on these cells (*n*=6, from 3 independent experiments). **A-C)** Data show the mean ± SEM.

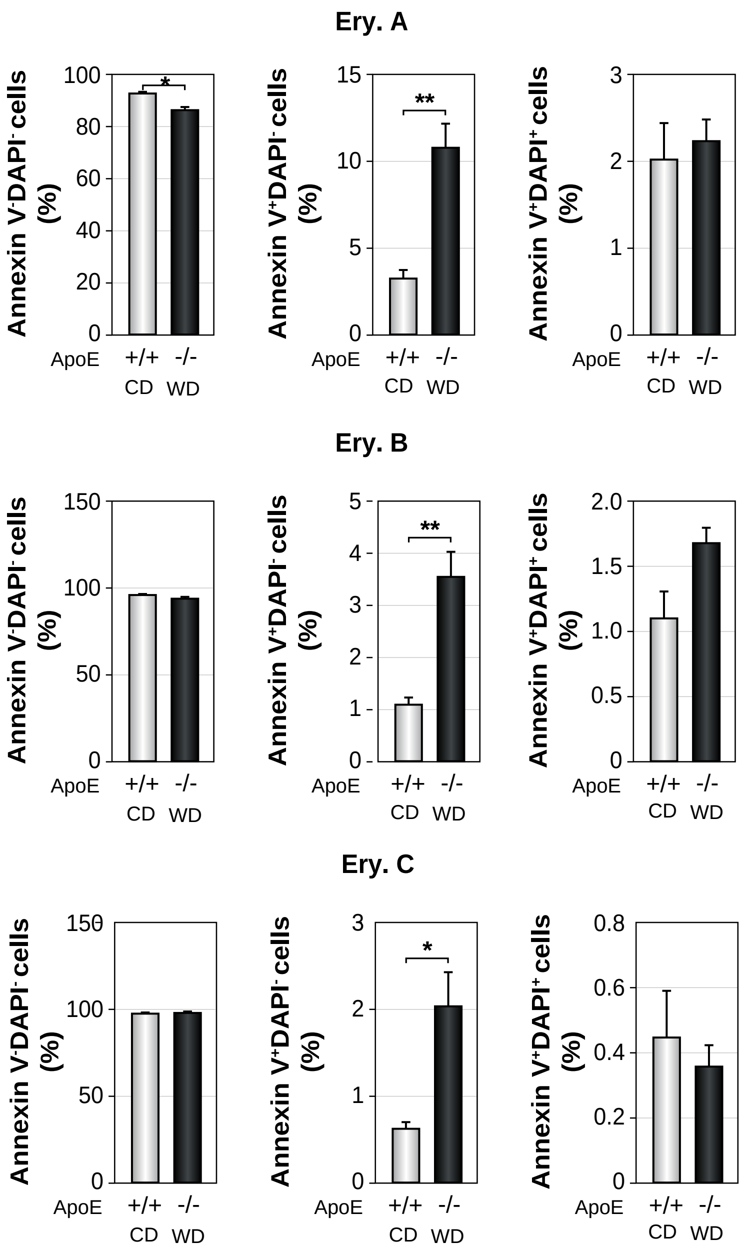

Supplementary Figure 5. Analysis of apoptosis among Ery. A, Ery. B and Ery. C from BM of control and atherogenic mice. Percentage of Annexin V^-DAPI^-, Annexin V^+DAPI^- and Annexin V^+DAPI^+ cells among BM Ery. A, Ery. B, and Ery. C from $ApoE^{+/+}$ mice fed a with CD and $ApoE^{-/-}$ mice fed with a WD for 13 weeks**.** Graphs show means ± SEM (*n*=5, from 2 independent experiments). Two-tailed Student's *t*-tests were used for comparisons of two groups. **p*<0.05, ***p*<0.01.

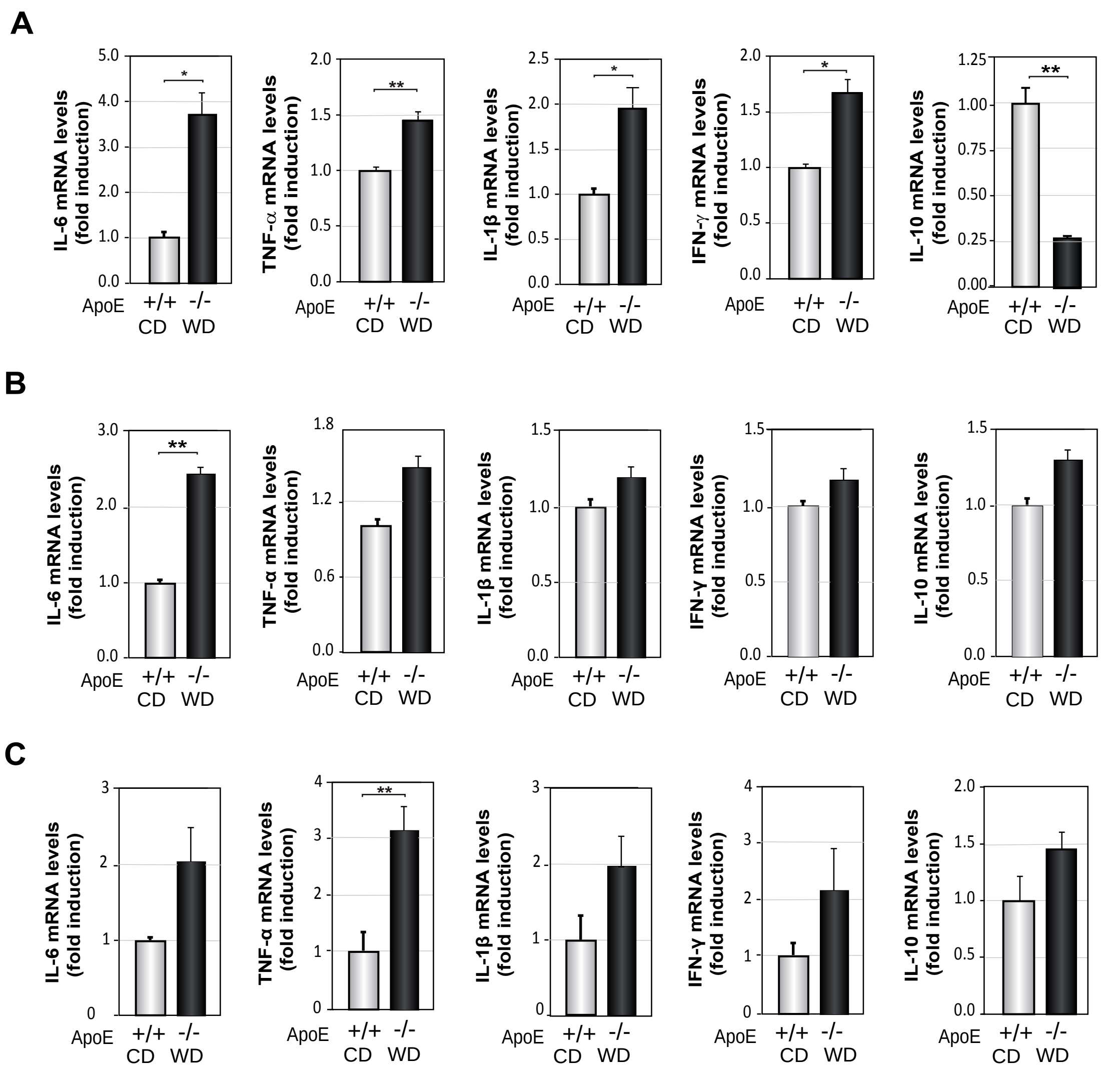

Supplementary Figure 6. Expression of cytokines in spleen, BM and liver of atherogenic and control mice. RNA from the spleen and BM of ApoE$^{-/-}$ mice fed a WD for 13 weeks and control mice was isolated and the expression levels of IL-6, TNF-α, IL-1β, IFN-γ and IL-10 in spleen (**A**), BM (**B**), and liver (**C**). **A-C)** Data show the mean ± SEM (n=7-8, from 3 independent experiments). Two-tailed Student's *t*-tests were used for comparisons between two groups.**p*<0.05, ***p*<0.01, ****p*<0.001.

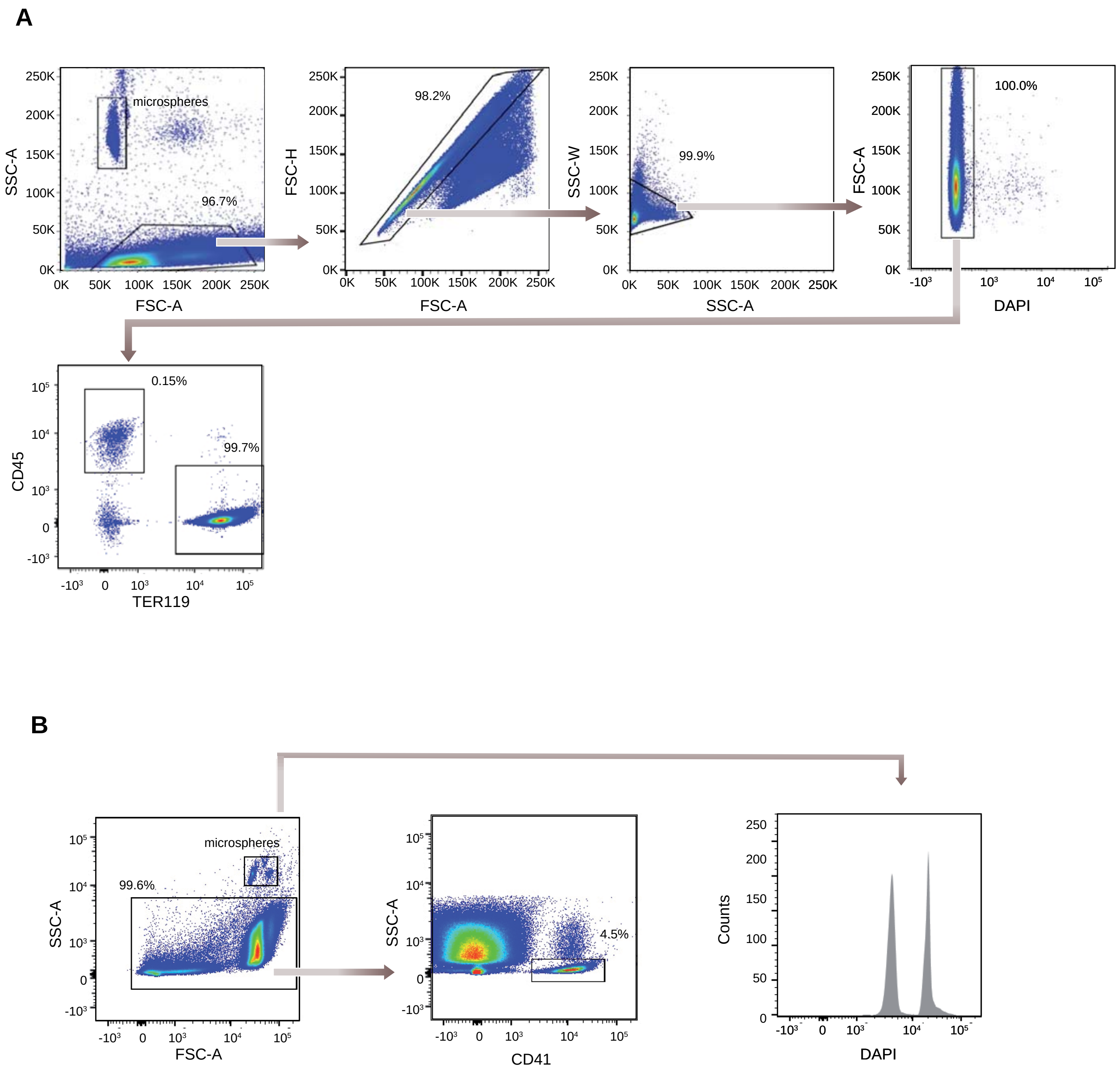

Supplementary Figure 7. Gating strategy for circulating RBC and platelets. A) Single live circulating cells were plotted for CD45 and Ter119. **B)** Events were plotted for SCC-A log and FCS-A log. Events below the $5x10^3$ SCC-A value were split based on their expression of CD41 and SCC-A value.

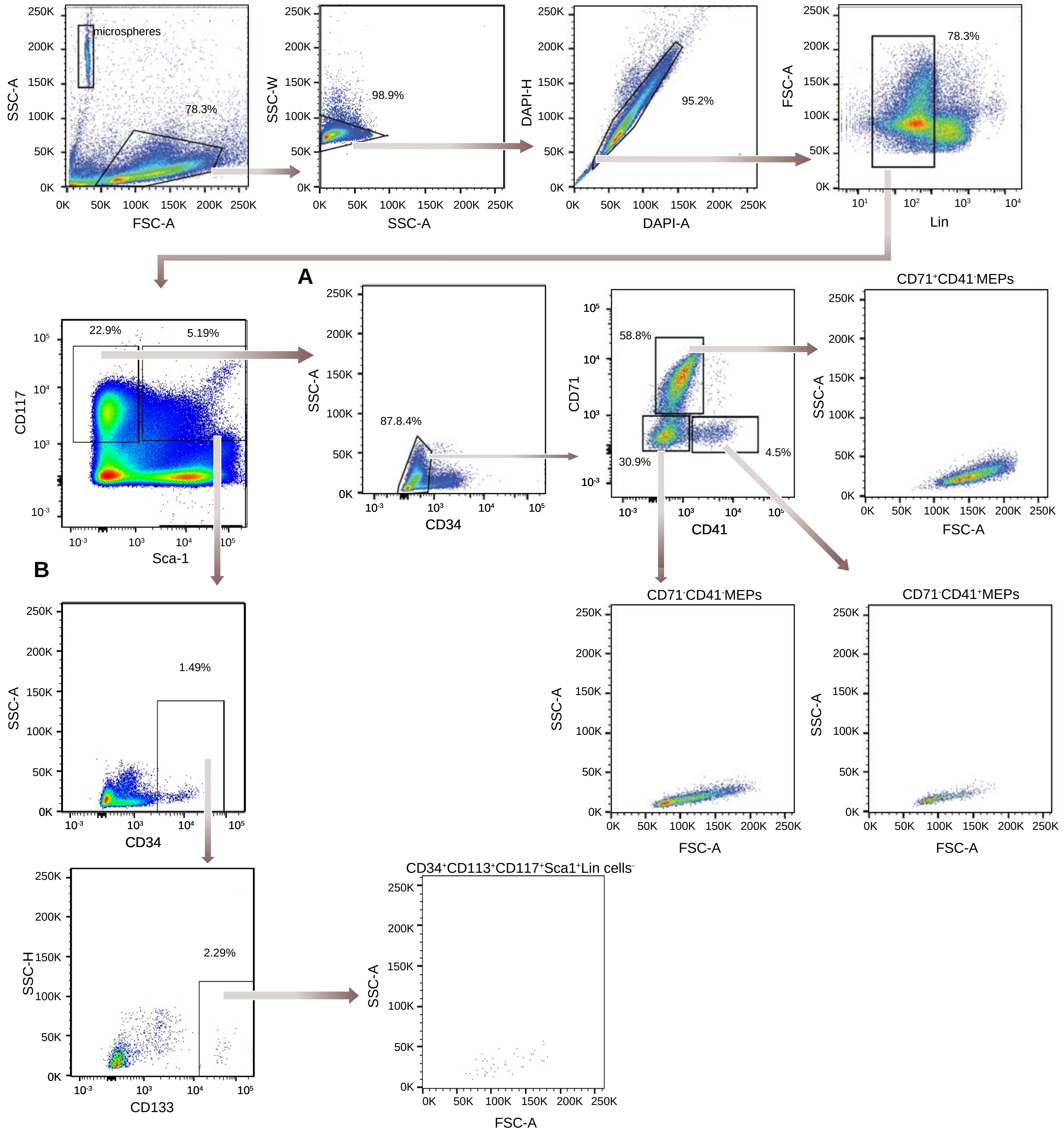

Supplementary Figure 8. Gating strategy for, CD71^{+}CD41^{-}MEPs, CD71^{-}CD41^{+}MEPs, and CD71^{-}CD41^{-}MEPs and CD34^{+}CD133^{+}CD117^{+}Sca1^{+}Lin^{-} cells. Lin^{-} magnetic-activated sorted single splenic cells were plotted for FSC-A and LIN. Subsequently, Lin^{-} cells were further gated on CD117 and Sca-1 expression. **A)** CD117^{+}Sca-1^{-} cells were further plotted for SSC-A and CD34. CD34^{-} cells were subdivided based on their CD41 and CD71 expression. **B)** CD117^{+}Sca-1^{+} cells were further plotted for SSC-A and CD34. CD34^{+} cells were subdivided based on their CD133 expression.

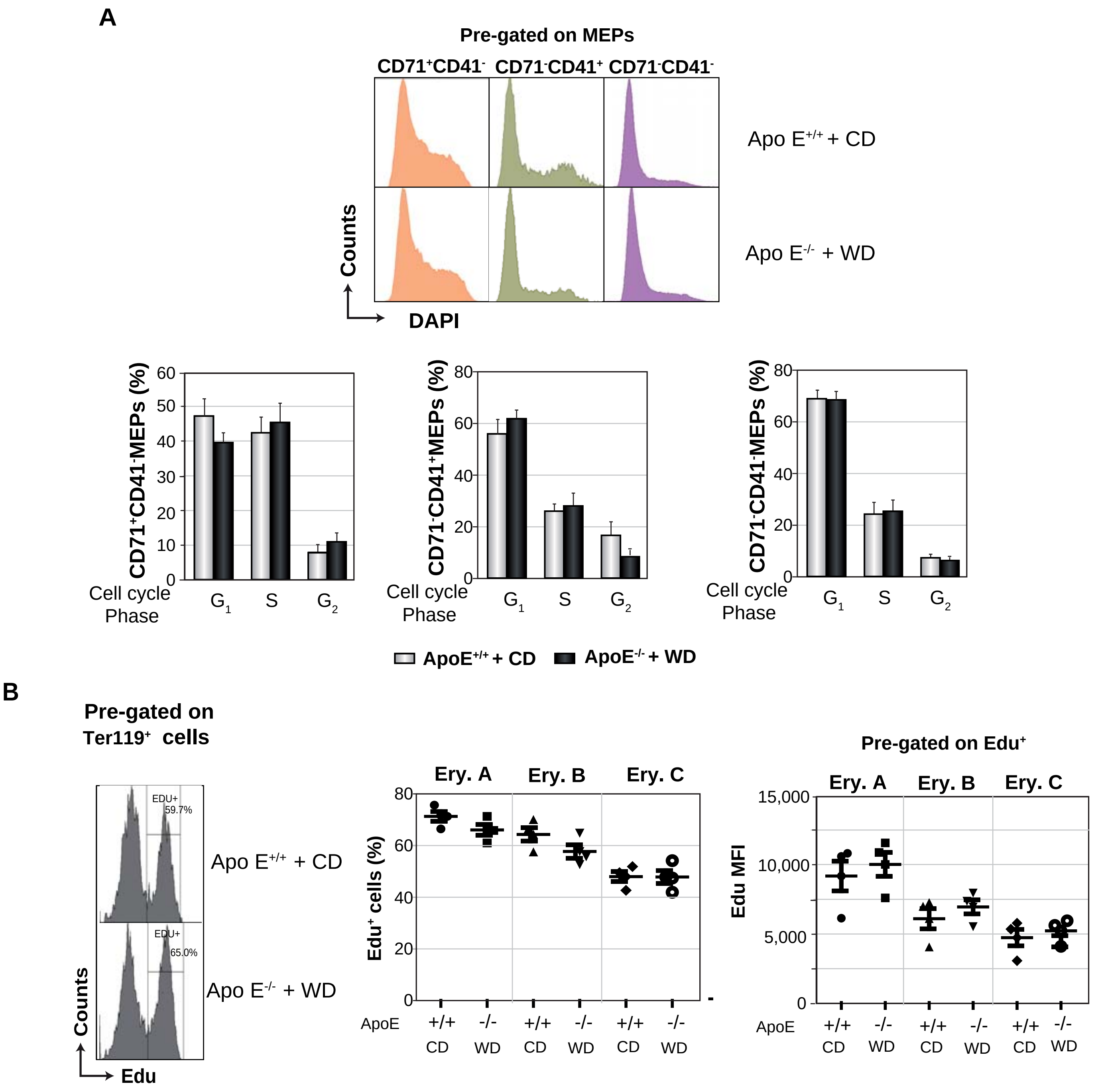

Supplementary Figure 9. Cell cycle progression of splenic CD71$^+$CD41$^-$MEPs, CD71$^-$CD41$^+$MEPs and CD71$^-$CD41$^-$MEPs from control and atherogenic mice. A) Lin$^-$ magnetic-activated sorted cells from the spleen of ApoE$^{+/+}$ mice fed with a CD and ApoE$^{-/-}$ mice fed with a WD for 13 weeks were surface stained, permeabilized and incubated with DAPI. CD71$^+$CD41$^-$ MEPs, CD71$^-$CD41$^+$MEPs and CD71$^-$CD41$^-$MEPs were further distributed according to their DAPI expression. Representative histograms depict the cell cycle distribution of CD71$^+$CD41$^-$ MEPs, CD71$^-$CD41$^+$MEPs and CD71$^-$CD41$^-$MEPs. The lower panels show the percentage of cells in the different phases of the cell cycle. Data from at least 3 independent experiments performed in duplicate are shown. **B)** Another set of mice described in A received an intraorbital injection of Edu and 4 hours later were killed. Splenic cells were surface-stained, permeabilized, and subjected to the Click-iT® reaction. Histograms depict a representative analysis of Edu$^+$ cells among the splenic Ter119$^+$ cell population. The graphs on the right show the percentage of the splenic Ery. A, Ery. B, and Ery. C cells positive for Edu and the mean fluorescence intensity (MFI) of Edu on these cells. **A,B)** Data show the mean ± SEM from two independent experiments performed at least in duplicate are presented.

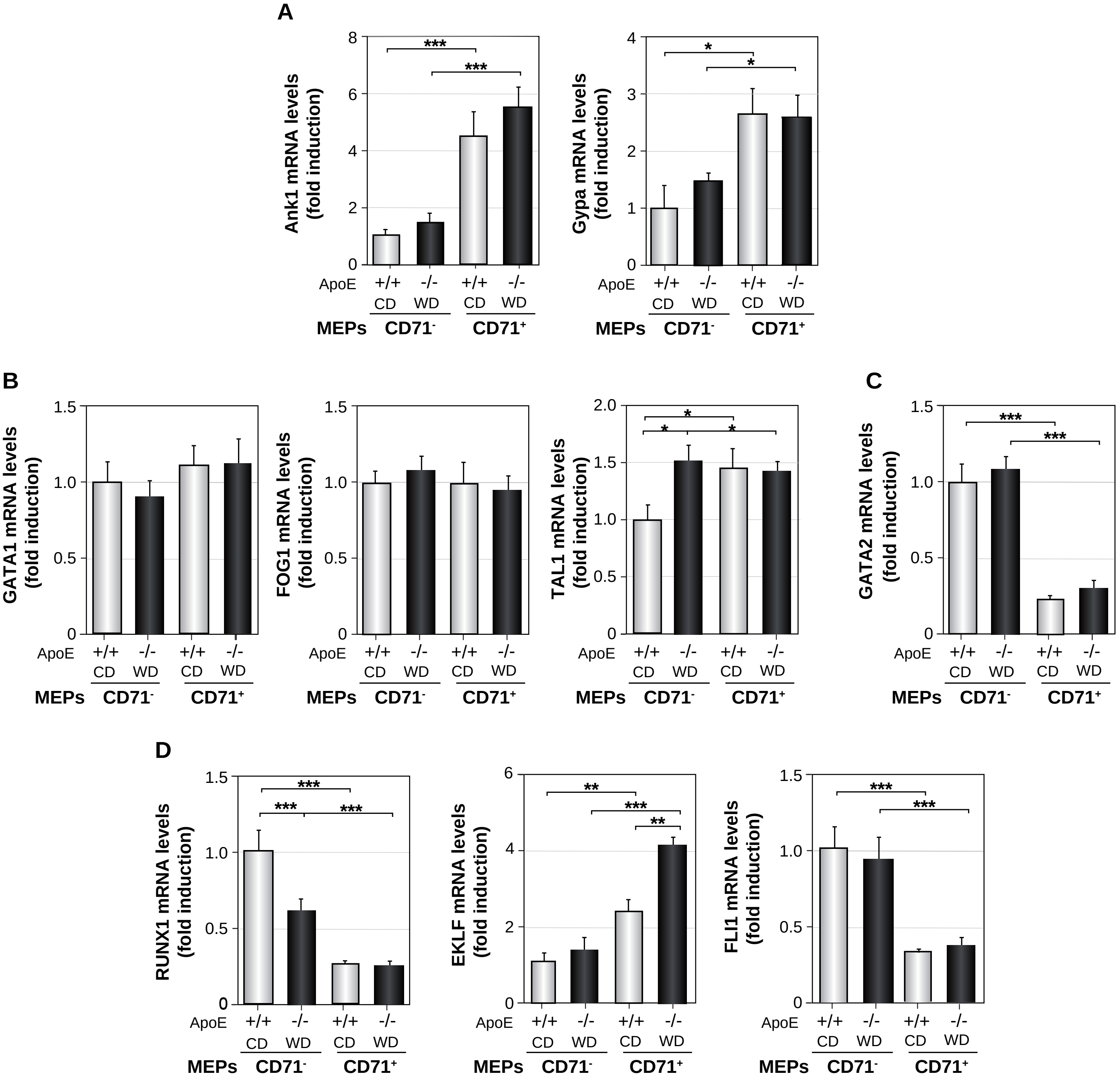

Supplementary Figure 10. Expression of key genes involved in the erythroid-megakaryocytic cell lineages bifurcation in splenic CD71^{+}CD41^{-}MEPs and CD71^{-}CD41^{-} MEPs from control and from atherogenic mice. Splenic CD71^{+}CD41^{-}MEPs and CD71^{-}CD41^{-} MEPs from ApoE$^{+/+}$ mice fed a CD and from ApoE$^{-/-}$ mice fed a WD for 13 weeks were sorted, and the expression of different genes involved in erythroid-megakaryocytic bifurcation of MEPs was analysed by RT-qPCR. **A)** Ankyrin1 (ANK1) and Glycophorin A (GYPA) mRNA levels (n=6-3, from 3-2 independent experiments). **B)** GATA1, FOG1 and TAL1 mRNA levels. **C)** GATA2 mRNA levels. **D)** RUNX1, EKLF and FLI1 mRNA levels. **B-D)** n=7-9, from 3-4 independent experiments. **A-D)** Data are expressed relative to the levels obtained in CD71^{-}CD41^{-}MEPs from control mice. One-way ANOVA with Bonferroni correction was used, to compare all pairs of columns between groups. The data show the significant differences between the two cells types of ApoE$^{+/+}$ mice fed a CD or of ApoE$^{-/-}$ mice fed with a WD, and between the same type of cells of ApoE$^{+/+}$ mice fed with a CD and of ApoE$^{-/-}$ mice fed a WD. *p<0.05, **p<0.01, ***p<0.001.

SUPPLEMENTARY TABLE S1

Mice	Cholesterol levels (mg/dl)
$ApoE^{+/+}$ +CD	104 +/- 3.8
$ApoE^{+/+}$ +WD	197 +/- 11
$ApoE^{-/-}$ +CD	285 +/- 24
$ApoE^{-/-}$+WD	1052 +/- 162

Male apolipoprotein $E^{+/+}$ ($ApoE^{+/+}$) and $ApoE^{-/-}$ C57BL/6J mice were fed a CD throughout or were switched to a WD, S167-EO12, high fat +7.5 g/kg cholesterol , Sniff Spezialdëten GmbH) at 10 week of age and maintained on this WD for 13 weeks (n=3) Cholesterol circulating levels were measured using commercial kits for total cholesterol (Reflotron, Roche Diagnostics, Mannheim Germany)

SUPPLEMENTARY TABLE S2

Antibody	Fluorocrome	Clone	Company
B220	PE, PerCP-Cy5.5	RA3-6B2	eBioscience
CD115	PE-Cy7	130-103-962	Miltenyi Biotec
CD117	APC	2B8	BD-Bioscience
CD133	PE	315-2C11	Biolegend
CD11b	FITC	130-081-201	Miltenyi Biotec
CD11b	APC-Cy7	MI-70	BD-Bioscience
CD11c	PE	N418	eBioscience
CD16/32	PE	93	Biolegend
CD172α	eFluor®710	P84	eBioscience
CD24	bio vright 510	M1/69	Biolegend
CD34	FITC	130-105-831	Miltenyi Biotec
CD41	PerCP-Cy5.5	MWReg30	Biolegend
CD45	PE-Cy7	30-F11	Invitrogen
CD47	APC	miap301	Biolegend
CD71	FITC, PE	RI7217	Biolegend
CD8a	APC	53-6.7	Biolegend
F4/80	APC	BM8	Biolegend
F4/80	bio vright 421	T45-2342	BD Bioscience
MHCII	super bright 600	M5/114.15.2	eBioscience
LIN	eFluor® 450	88-7772-72	eBioscience
LIN	FITC	22-7770-72	eBioscience
SCA-1	PE-Cy7	130-106-220	Miltenyi Biotec
TER 119	PE, APC	ter	Biolegend

SUPPLEMENTARY TABLE S3

Gene	Foward primer (5'-3')	Reverse primer (5'-3')
ANK1	ATTAACACCTGTAACCAGAACGG	GGGCATTGACATTGGCTCCA
BMP4	ATTCCTGGTAACCGAATGC	CCGGTCTCAGGTATCAAAC
EKLF	AGACTGTCTTACCCTCCATCAG	GGTCCTCCGATTTCAGACTCAC
EPO	ACTCTCCTTGCTACTGATTC	ATCGTGACATTTTCTGCCTC
FLI-1	ATGGACGGGACTATTAAGGAGG	GAAGCAGTCATATCTGCCTTGG
FOG-1	AGGAAACAGAGCAATCCCCG	CAGGTGGGCTCACATCTTCT
GATA-1	TGGGGACCTCAGAACCCTTG	GGCTGCATTTGGGGAAGTG
GATA-2	CACCCCGCCGTATTGAATG	CCTGCGAGTCGAGATGGTTG
GDF15	CTGGCAATGCCTGAACAACG	GGTCGGGACTTGGTTCTGAG
GYPA	GAATGCCGTCACCAATTCAAC	AGTTCCGATAATCCCTGCCAT
HEPCIDIN	TTGCGATACCAATGCAGAAG	TGCAACAGATACCACACTGG
HPRT	TCAGTCAACGGGGGACATAAA	GGGGCTGTACTGCTTAACCAG
IFN-γ	ATCTGGAGGAACTGGCAAA	TTCAAGACTTCAAAGAGTCTGAGGTA
IL-1β	AGTTGACGGACCCCAAAAG	AGCTGGATGCTCTCATCAGC
IL-6	GCTACCAAACTGGATATAATCAGGA	CCAGGTAGCTATGGTACTCCAGAA
IL-10	AGCCTTATCGGAAATGATCCAGT	GGCCTTGTAGACACCTTGGT
RUNX-1	GATGGCACTCTGGTCACCG	GCCGCTCGGAAAAGGACAA
TAL-1	CACTAGGCAGTGGGTTCTTTG	GGTGTGAGGACCATCAGAAATCT
TNF-α	GCCAACATCCCTACCTCTCC	CCCCAGGGCAAAGGTAAT
18S	CCAGTAAGTGCGGGTCATAAGC	CCTCACTAAACCATCCAATCGG
